# Assessing motivation to change in eating disorders: a systematic review

**DOI:** 10.1186/2050-2974-1-38

**Published:** 2013-10-10

**Authors:** Katrin Hoetzel, Ruth von Brachel, Lena Schlossmacher, Silja Vocks

**Affiliations:** 1 Department of Psychology, Clinical Psychology and Psychotherapy, Osnabrück University, Knollstraße 15, 49069, Osnabrück, Germany; 2 Department of Psychology, Clinical Child and Adolescent Psychology, Ruhr-University Bochum, Universitätsstraße 150, 44801, Bochum, Germany

**Keywords:** Anorexia nervosa, Assessment, Bulimia nervosa, Interview, Motivation to change, Questionnaire

## Abstract

**Background:**

Patients with anorexia and bulimia nervosa are often ambivalent about their eating disorder symptoms. Therefore, a lack of motivation to change is a frequent problem in the treatment of eating disorders. This is of high relevance, as a low motivation to change is a predictor of an unfavourable treatment outcome and high treatment dropout rates. In order to quantify the degree of motivation to change, valid and reliable instruments are required in research and practice. The transtheoretical model of behaviour change (TTM) offers a framework for these measurements.

**Objective:**

This paper reviews existing instruments assessing motivation to change in eating disorders.

**Method:**

We screened *N* = 119 studies from the databases Medline and Psycinfo found by combinations of the search keywords ‘eating disorder’, ‘anorexia nervosa’, ‘bulimia nervosa’, ‘motivation’, ‘readiness to change’, ‘assessment’, ‘measurement’, and ‘questionnaire’.

**Results:**

Ultimately, *n* = 15 studies investigating psychometric properties of different assessment tools of motivation to change in eating disorders were identified. Reviewed instruments can be divided into those assessing the stages of change according to the TTM (6 instruments) and those capturing decisional balance (3 instruments). Overall, the psychometric properties of these instruments are satisfactory to good.

**Discussion:**

Advantages, disadvantages, and limitations of the reviewed assessment tools are discussed. So far, the TTM provides the only framework to assess motivation to change in eating disorders.

## Introduction

Patients with anorexia and bulimia nervosa are known to be ambivalent about their eating disorder symptoms [1]. On the one hand, the eating disorder is perceived as a burden, but on the other hand, it also provides reasons to hold on to it [2]. Consequently, patients with eating disorders often display a low motivation to change [3-5]. This low motivation to change is often viewed as the cause for the high dropout rates or lack of engagement which are major problems in the treatment of anorexia and bulimia nervosa [6,7]. Further indications of the high clinical relevance of motivation to change in eating disorders are provided by several studies suggesting a positive association between a high motivation to change and several desirable clinical indices (such as continuing treatment, decreases in eating pathology, increases in weight, and weight maintenance) [8-11]. Based on these results, several studies have been conducted in order to assess the effects of interventions aiming at an enhancement of motivation to change in eating disorders (for an overview, see [12]).

Given the high clinical relevance of motivation to change in eating disorders and the great interest in investigating this field, there is a need for appropriate psychometric assessment tools. Such tools could ensure a more uniform and valid methodology for future research [13]. Furthermore, as patients’ and clinicians’ perceptions of motivation to change have been shown to differ significantly [14] and clinicians’ ratings have been shown to be unrelated to any outcome measures [15], specific and valid assessment tools are required not only for science, but also for clinical practice. Such instruments enable insights, for instance, into the patient’s perception of benefits and burdens of a change [2] and in this respect help therapists to optimise the selection of treatment strategies for each patient.

Several measures to assess motivation to change have been developed, all of which are rooted in the transtheoretical model of behaviour change (TTM [16]), which represents the most common theoretical framework in this research area. The TTM offers an explanation for motivation to change in general and has been applied to several health problems and mental disorders, including eating disorders [13]. It defines ‘stages’ of readiness for change, which occur through a series of six motivational stages (i.e., precontemplation, contemplation, preparation, action, maintenance, and termination) and are characterised by various degrees of involvement in the therapeutic process. Furthermore, the TTM comprises a theory on decision making [17], which is considered to be a necessary process in order to progress through the stages. The decision making process depends on the balance of perceived pros and cons for a problem behaviour, which is also known as decisional balance (for a comprehensive review of the TTM, see [16]). Derived from these theoretical assumptions, instruments on motivation to change can be categorised into two groups: those assessing the stages of change and those capturing decisional balance.

To summarise, it can be stated that motivation to change is an important aspect of the treatment of eating disorders. The application of evaluated instruments which assess motivation to change in eating disorders is urgently required for research and practice. However, as yet to our knowledge, no review on instruments that measure motivation to change in anorexia and bulimia nervosa has been published. Therefore, the aim of the present paper is to review the existing instruments assessing motivation to change in eating disorders regarding their psychometric properties and to provide a critical evaluation of these tools.

## Method

We selected studies from the databases Medline and Psycinfo by searching for the combined keywords ‘eating disorder’, ‘anorexia nervosa’, ‘bulimia nervosa’, ‘motivation’, ‘readiness to change’, ‘assessment’, ‘measurement’, ‘questionnaire’, and ‘interview’ from February to March 2013. Five articles were added which were retrieved from references of included articles or were known of by the authors from other sources. Papers investigating the psychometric properties of assessment devices for motivation to change in eating disorders were included in this review. Furthermore, the papers had to be published in a peer-reviewed journal in the English language. We only included instruments for which information both on reliability and validity was published. Papers on instruments for which only information concerning validity was available were excluded, as reliability is considered as the precondition for all forms of validity [18]. Moreover, only studies which used eating disorder-specific questionnaires for the assessment of motivation to change were examined.

## Review

**Figure 1:**
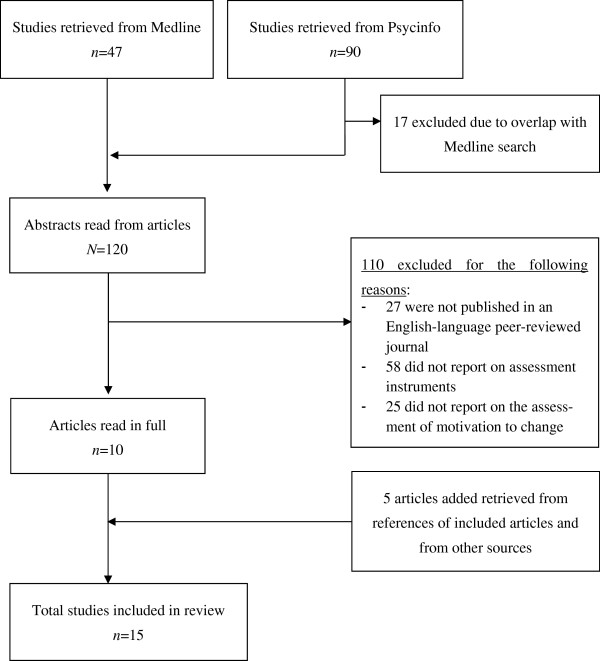
**QUORUM diagram showing results of the literature search** ***.***

Figure 1 presents a QUORUM diagram of the literature search. The final set of papers comprised *n* = 15 papers.

Table 1 presents the identified papers on instruments specifically assessing motivation to change in eating disorders. The identified instruments differ according to whether they measure the stages of change or decisional balance. Furthermore, most of the instruments are questionnaires, while one is an interview. In the following section, the interview assessing the stages of change is introduced first; following this, questionnaires measuring the stages of change are presented. Finally, decisional balance scales are presented.

**Table 1:** Instruments measuring motivation to change in eating disorders

**Name of instrument**	**Validation study**	**Format**	**Subscales**	**Sample**	**Reliability**	**Validity**
Assessment of the stages of change according to transtheoretical model of behaviour change						
*Readiness and Motivation Interview for eating disorders* (*RMI*)	[19,20]	Semi-structured interview	Four subscales:	*N* = 99 AN, BN, < EDNOS adults (inpatients)	Interrater agreement:	CV: Significant correlations with URICA and PCQ
			‘Precontemplation’, ‘Contemplation’, ‘Action’, ‘Internality’		95.6% - 97.4%	DV: Non-significant correlations with age, socio-economic status, BMI, and social desirability^a^
					*α* = .63 - .84	PV: Prediction of anticipated difficulty of completing tasks related to eating disorder recovery^b^, completion of recovery activities^c^, decision to enrol in treatment, and dropout
	[21]		Two subscales:	*N* = 65 AN, BN, < EDNOS adolescents (inpatients and outpatients)	Interrater agreement:	CV: Significant correlations with URICA and PCQ
			‘Precontemplation’, ‘Action’ (as the internal consistency for ‘Contemplation’ and ‘Internality’ was unacceptably low)		90.3% - 97.9%	DV: Non-significant correlations with age, socio-economic status, BMI, and social desirability^d^
					*α* = .19 - .79	PV: Significant correlations with anticipated difficulty of completing tasks related to eating disorder recovery^b^; prediction of completion of recovery activities^c^
*Anorexia Nervosa Stages of Change Questionnaire* (*ANSOCQ*)	[22]	Self-report questionnaire (20 items)	Each item is regarded separately and a total score can be calculated.	*N* = 71 AN adults and adolescents (inpatients)	*r*_*tt*_ = .89 *α* = .90	CV: Significant correlations with URICA; negative correlations of ANSOCQ total score with EDI-2
						DV: Non-significant correlations of ANSOCQ total score with social desirability in adults^a^, but positive correlations with social desirability in adolescents^e^
						PV: Prediction of weight gain during treatment by ANSOCQ total score; significant correlations between ANSOCQ total score at commencement of treatment and EDI-2 at discharge
	[23]			*N* = 44 AN adults and adolescents (inpatients)		CV: Positive correlations of ANSOCQ total score with self-efficacy^f^, DB subscale ‘Burden’, and negative correlations with DB subscales ‘Benefits’ and ‘Avoidance Coping’, and CSS total score
	[24]			*N* = 70AN adolescents (inpatients and outpatients)	*r*_*tt*_ = .90*α* = .94	CV: Negative correlations of ANSOCQ total score with EDI-2 and BDI
						
*Bulimia Nervosa Stages of Change Questionnaire* (*BNSOCQ*)	[25]	Self-report questionnaire (20 items)	Each item is regarded separately and a total score can be calculated.	*N* = 30BN adolescents (inpatients)	*r*_*tt*_ = .94*α* = .93	CV: Negative correlations of BNSOCQ total score with BDI-2 and EDI-2
						DV: Non-significant correlations of the BNSOCQ total score with BMI and illness duration, but positive correlations with age
*Eating Disorders Stage of Change* (*EDSOC*)	[26]	Self-report questionnaire (8 items)	Each symptom domain is regarded separately.	*N* = 145AN, BN, < EDNOS adults and adolescents (inpatients and outpatients)	*r*_*tt*_ = .55 - 1.00*α* = .33 - .78	CV: Positive correlations of ‘Restrict’, ‘Diet Pill Use’, and ‘Fast’ with URICA
						DV: Non-significant correlations with BMI, but negative correlations of ‘Fast’, ‘Restrict’, ‘Purge’, ‘Laxative Use’, and ‘Diet Pill Use’ with BSQ and positive correlations of ‘Purge’, ‘Laxative Use’ and ‘Diet Pill Use’ with age
*Motivational Stages of Change for Adolescents Recovering from an Eating Disorder* (*MSCARED*)	[27]	Questionnaire filled out together with an interviewer	Motivation for change and, if the youth is in action or maintenance phase, actions currently undertaken are rated.	*N* = 34AN, BN, < EDNOS adolescents (outpatients)	*r*_*tt*_ = .92	CV: Positive correlations of the youth’s self-reported stage of change with the interviewer’s and the mother’s; lower EDI-2 and CDI scores in higher phases
					(*N* = 16)	DV: Non-significant correlations with the diagnostic category, and with initial or final BMI levels
*Readiness and Motivation Questionnaire (RMQ)*	[28]	Self-report questionnaire (5 items for each of 12 symptom domains)	Two motivational stage scores (‘Precontem-plation’, ‘Action’) for each of four symptom domains; locus of control (‘Internality’, ‘Confidence’)	*N* = 207AN, BN, < EDNOS adults (outpatients)	*r*_*tt*_ = .62 - .81*α* = .55 - .80	CV: Positive correlations of EDI with ‘Precontemplation’ and negative correlations with ‘Action’ and ‘Confidence’. Significant correlations with URICA and RMI.
						DV: Non-significant correlations with BMI, self-efficacy^c^, and social desirability^a^; negative correlations of ‘Confidence’ with age
						PV: Significant correlations with the anticipated difficulty of recovery activities^f^ and completion of recovery activities^d^
Decisional Balance Scales						
*Decisional Balance Scale for Anorexia Nervosa* (*DB*)	[29]	Self-report Likert scale (72 items)	Three subscales:	*N* = 246AN adults (inpatients and outpatients)	*r*_*tt*_ = .64 - .71*α* = .88 for each of the three subscales	
			‘Burdens’, ‘Benefits’, ‘Functional Avoidance’			
	[30]			*N* = 80AN < EDNOS adults (outpatients)		CV: Positive correlations of ‘Burdens’ with PCQ; non-significant correlations of ‘Benefits’ and ‘Functional Avoidance’ with PCQ
						DV: Non-significant correlations with social desirability^a^, socio-economic status, and BMI; significant correlations of ‘Functional Avoidance’ with age
*Pros and Cons of Anorexia Nervosa scale* (*P-CAN*)	[31]	Self-report Likert scale (50 items)	Six pro-scales:	*N* = 233AN adults (inpatients and outpatients)	*r*_*tt*_ = .60 - .85*α* = .52 - .78	CV: Positive correlations of P-CAN pro-scales ‘Appearance’, ‘Communicate Emotions/Distress’, ‘Fitness’, and ‘Safe/Structured’ with EDI; negative correlations of the P-CAN con-scale ‘Hatred’ with EDI
			‘Safe/Structured’; ‘Appearance’; ‘Fertility/Sexuality’; ‘Fitness’; ‘Communicate Emotions/Distress’; ‘Special/Skill’			DV: Non-significant correlations of the P-CAN subscales with BMI
			Four con-scales:			
			‘Trapped’; ‘Guilt’; ‘Hatred’; ‘Stifles Emotions’			
	[32]			*N* = 48AN adolescents (inpatients and outpatients)	*α* = .73 - .97	CV: Positive correlations of the P-CAN pro-scales ‘Communicate Emotions/Distress’, ‘Special’, ‘Safe/Structured’ with EDE-Q global score; positive correlations of P-CAN con-scales with EDE-Q total score
						DV: Non-significant correlations of P-CAN subscales with BMI
*Pros and Cons of Eating Disorders scale* (*P-CED*)	[33]	Self-report Likert scale (72 items)	Subscales of P-CAN and four additional ones: pro-scales:	*N* = 202past or current diagnosis of AN or BN adults (outpatients)	None reported	DV: Significant differences between patients with AN and BN on P-CED subscales ‘Safe/Structured’ (AN > BN), ‘Special/Skills’ (AN > BN), ‘Fitness’ (AN > BN), ‘Fertility/Sexuality’ (AN > BN), ‘Eat but Stay Slim’ (AN < BN), ‘Guilt’ (AN > BN)
			‘Boredom’; ‘Eat but Stay Slim’; con-scales: ‘Negative Self-Image’; ‘Weight and Shape’			

*Note.* In all cases, test-retest reliability was measured after approximately one week. The following abbreviations are used: AN = anorexia nervosa; BDI II = Beck Depression Inventory [34]; BN = bulimia nervosa; BSQ = Body Shape Questionnaire [35]; CDI = Children’s Depression Inventory [36]; CSS = Concerns about Change Scale [37]; CV = convergent validity; DV = divergent validity; EDE-Q = Eating Disorder Examination Questionnaire [38]; EDI = Eating Disorder Inventory [39]; EDI-2 = Eating Disorder Inventory-2 [40]; EDNOS = eating disorder not otherwise specified; PCQ = Processes of Change Questionnaire [41]; PV = predictive validity; *r*_*tt*_ = test-retest reliability.

^a^measured with Balanced Inventory of Desirable Responding [42].

^b^measured with Anticipated Difficulty of Recovery Activities [21,24].

^c^measured with Completion of Recovery Activities [21,24].

^d^measured with Marlowe-Crowne Social Desirability Scale [43].

^e^measured with Children’s Social Desirability Scale [44].

^f^measured with Self-Efficacy Scale for AN [22].

### Interview assessing the stages of change

The *Readiness and Motivation Interview* (*RMI*[19,20]) is a semi-structured interview which contains several questions to assess motivation to change. The stages of change are measured according to four different symptom domains of the eating disorder (i.e., restriction, cognition, bingeing, and compensatory behaviour). If a patient is in the stage of action, the locus of control is also rated. As displayed in Table 1, reliability and validity are very good. In addition to adults, the *RMI* has also been used in a younger population of 12-18-year-old girls with eating disorders [21].

### Questionnaires assessing the stages of change

Further identified measurements of the stages of change according to the TTM are the *Anorexia Nervosa Stages of Change Questionnaire* (*ANSOCQ*[22,23]), the *Bulimia Nervosa Stages of Change Questionnaire* (*BNSOCQ*[25]), the *Eating Disorders Stages of Change Questionnaire* (*EDSOCQ*[26]), the *Motivational Stages of Change for Adolescents Recovering from an Eating Disorder* (*MSCARED*[27]), and the *Readiness and Motivation Questionnaire* (*RMQ*[28]). The *ANSOCQ*, *BNSOCQ*, *EDSOCQ*, and *RMQ* assign a stage of change for each symptom domain of the eating disorder (e.g., gaining weight, importance of body shape and weight, fear of fatness). According to this conceptualisation, a person might, for example, be in the contemplation stage regarding her bingeing behaviour, but can simultaneously be in the precontemplation stage concerning her attempt to remain very thin. Unlike these four questionnaires, the *MSCARED* assesses the stages of change for adolescents currently recovering from an eating disorder in a global manner. With this measurement, the adolescent is allocated to one stage of change for the motivation to change the eating disorder as a whole.

The *ANSOCQ* and *BNSOCQ* were designed for specific diagnostic groups, i.e., patients with anorexia and bulimia nervosa, respectively. The *EDSOCQ*, *MSCARED*, and *RMQ* can be applied to patients with anorexia nervosa, bulimia nervosa, and eating disorder not otherwise specified. Psychometric properties of the *ANSOCQ* and *EDSOCQ* have been analysed both in adults and adolescents [22-24,26], while the psychometric properties of the *BNSOCQ* and *MSCARED* have been evaluated for adolescents only [25,27] and those of the *RMQ* for adults [28] only. As displayed in Table 1, reliability and validity of these four questionnaires are generally good.

### Decisional balance scales

The literature search revealed two decisional balance scales for anorexia nervosa, the *Decisional Balance Scale for Anorexia Nervosa* (*DB*[29,30]) and the *Pros and Cons of Anorexia Nervosa* (*P-CAN*[31]). The *DB* assesses both the perceived ‘Benefits’ and ‘Burdens’ of anorexia nervosa (e.g., gains or losses for the self and significant others, self-approval, self-disapproval, approval and disapproval of others) as well as ‘Functional Avoidance’ (e.g., how the disorder prevents the individual from dealing with emotions). It was adapted from a scale by Rossi et al. [45], while the *P-CAN* was derived from an analysis of the themes endorsed by patients suffering from anorexia nervosa [46]. The *P-CAN* gives insight into the perceived pros and cons of the individual’s anorexia nervosa. As Table 1 shows, it has been successfully applied to both adult [31] and adolescent populations [32] and reliability as well as validity is sufficient.

To broaden the application of the *P-CAN* to individuals with bulimia nervosa, some subscales dealing with bingeing and purging were added to the *Pros and Cons of Eating Disorders Scale* (*P-CED*) and the term ‘anorexia nervosa’ was substituted by ‘eating disorder’ [33]. Although information on the reliability of the *P-CED* has not been published, it is nevertheless included here, as its items are nearly identical to the *P-CAN*, whose reliability is considered sufficient.

## Discussion and conclusion

The aim of the present paper was to review assessment tools for motivation to change in eating disorders. 15 studies on nine different assessment tools for motivation to change in eating disorders were included. We identified *n* = 6 instruments assessing motivation to change according to the stages of change and *n* = 3 instruments capturing decisional balance.

Those instruments measuring the stages of change provide different types of assessments, measuring the stages either globally, i.e., motivation to change the eating disorder as a whole (e.g., *MSCARED*), or in a symptom-specific manner, i.e. motivation to change bingeing (e.g., *ANSOCQ* or *RMI*). However, due to the complexity regarding the different symptom domains of an eating disorder, it might be inappropriate to allocate a person to a single global stage [21,26,47], as a symptom-specific measure of the stages of change was found to explain more variance of a problem behaviour than a global-stage assessment [48]. For example, it was shown that the motivation to change bingeing behaviour as one symptom domain was uniformly high across individuals, while motivation to change dietary restriction as another domain was rather low [21,49]. These requirements are especially fulfilled by the interview *RMI*[19,20] and the questionnaires such as the *ANSOCQ*[22,23], *BNSOCQ*[25], *EDSOCQ*[26], and *RMQ*[28], which are more economical to apply. Thus, in some recent studies, possibly existing differences in specific symptom domains may have remained undetected due to the global assessment of motivation to change [3,50].

However, in some cases, there seems to be a disconnection between the approach of measuring motivation in a symptom-specific manner and the actual statistical analyses conducted with the medium over all heterogeneous symptom domains. Even though the items of the *ANSOCQ* and *BNSOCQ* assess multiple dimensions of motivation to change, there are no separate subscales providing scores for each symptom domain, and instead, a global total score is calculated. In the reviewed studies, this total score was also used to evaluate these questionnaires, which contradicts the original intention of a symptom-specific approach. Furthermore, as they comprise only one item per symptom domain, no subscales can be calculated and it is therefore not possible to determine some aspects of reliability, i.e., internal consistency. However, the *RMI* and its related questionnaire *RMQ* as well as the *EDSOCQ* constitute exceptions to these aspects, as the authors calculate separate scores for each subscale and provide information on the psychometric properties of each of the subscales. Nevertheless, the *ANSOCQ* and *BNSOCQ* are useful tools in practice for planning treatment, as they allow an insight into motivation concerning different eating disorder symptoms.

Measures provided in a questionnaire format are widespread as they provide an economical and reliable method of measurement. They are easy to administer, require little or no training, and are less time-consuming than interviews. However, they also have some disadvantages compared to interviews such as the *RMI*[28], especially concerning the validity in an ambivalent clientele like women with eating disorders [51]. The use of questionnaires to measure the stages of change has been criticised due to oversimplification and the danger of alienating patients [52]. To prevent misunderstandings while filling out questionnaires, and in order to build a more collaborative therapeutic relationship [53], an interview procedure like the *RMI* seems to be highly suitable if a time-consuming application is possible. However, most studies on motivation to change in eating disorders conducted to date exclusively applied questionnaires [4,9,50,54-57] and many [4,58] used the *University of Rhode Island Change Assessment Scale* (*URICA*[59,60]), which is not specifically addressed at patients with eating disorders but rather at problem behaviours in general. Moreover, the *URICA* can be criticised for measuring motivation to change a problem behaviour in general terms and not in a symptom-specific manner with each symptom domain being assessed separately [26]. For a more detailed investigation of motivation to change in eating disorders, it can be recommended that in future research, more precise symptom- and disorder-specific instruments should be used, such as the *RMI*, *RMQ*, and *EDSOCQ*.

One of the most important features of an instrument measuring motivation to change is probably that it predicts readiness to make changes. Concerning their predictive utility, the *RMI*, *RMQ*, and *ANSOCQ* have proven most beneficial, as their relative ability to predict clinical outcome temporally has received the best support so far. The *ANSOCQ* has proven to be a useful tool in this regard, as it predicts weight gain during treatment [22], while lower motivation scores on the *RMI* at baseline were associated with treatment dropout after 12–15 weeks [20]. Furthermore, both *RMI* and *RMQ* scores predicted completion of recovery activities over time [20,21,28]. Evidence has also been found concerning the prediction of clinical outcomes from a cross-sectional perspective for these three measures, while support for the predictive validity of all other reviewed instruments is so far lacking.

In spite of the current lack of predictive clinical utility of decisional balance scales, the potential role of these assessment tools in treatment planning should be nevertheless considered. The *DB*, *P-CAN*, and *P-CED* may be useful for therapists to gain an insight into the pros and cons of an eating disorder as perceived by the patient, especially concerning the maintenance of the disorder.

Research conducted to date suggests that there are qualitative differences between patients with anorexia and bulimia nervosa regarding their arguments for and against their disorder [1]. Moreover, patients suffering from anorexia nervosa seem to be less motivated for behavioural change than those with bulimia nervosa [4]. Thus, the diagnosis-specific questionnaires *ANSOCQ, DB, P-CAN,* and *BNSOCQ* are suitable for diagnostically homogeneous populations consisting of patients with anorexia or with bulimia nervosa. However, in order to compare the differences regarding the extent of motivation to change between patients with various eating disorder diagnoses, measurements such as the *EDSOCQ*, *MSCARED*, *RMI*, *RMQ*, or *P-CED* are useful tools. They are therefore more in line with the transdiagnostic model of eating disorders [61].

It has to be taken into consideration that all of the assessment tools reviewed here are based on the TTM. Although the TTM has been successfully applied to a number of health problems and there is a large body of research supporting its assumptions [13,62], it has been criticised concerning several aspects [63]: Due to the categorical nature of this model, critics point out that motivation to change might be better measured on a continuum rather than in distinct categories and that the stages do not constitute discrete categories because it is possible for individuals to be in more than one stage at the same time. However, to date, the research literature does not provide any alternative solution to this categorical model and the categorical assessment approach. In contrast to the theory based on categories, statistical analyses are sometimes not based on ordinal-scaled variables. Data are rather interpreted as interval-scaled instead, which acts on the assumption of a dimensional model and no distinct categories. If the TTM is not a valid model, the instruments derived from it may not be valid either. Thus, ideally, future research should develop dimensional approaches in measuring motivation to change as a continuum and compare them with previous tools, especially in terms of their validity. However, the TTM has proven to be useful in clinical research and practice [63], and significant relationships between initial stage of change and treatment outcome have been demonstrated in the eating disorders in several, but not all, studies [13].

Finally, this review is subject to the limitation that only published literature was included. It is not uncommon for unpublished and non-validated scales to be used for investigation purposes. These unpublished assessment tools were not reviewed here as it was not possible to systematically identify them through the literature search.

To conclude, the TTM, which has been criticised due to its categorical assumptions, offers the only theoretical framework so far for tools which assess motivation to change in eating disorders. Nevertheless, psychometric properties of the identified instruments are satisfactory to good. All assessment tools have specific benefits and burdens and the selection of a specific instrument depends on the context of assessment. Overall, the *RMQ* and *RMI* in particular fulfil important aspects of an instrument measuring motivation to change in eating disorders, as they measure the construct in a symptom-specific way, allow the calculation of subscales for different symptom domains, can be applied to heterogeneous groups, and provide predictive utility. The *RMQ*, as a questionnaire, is rather economical, less time-consuming, reliable, and easy to apply, while the *RMI*, as an interview, requires time and training but might be most valid and helpful in order to build a collaborative relationship for treatment.

## Competing interests

The authors declare that they have no competing interests.

## Authors’ contributions

All authors contributed to the development of this review and drafted the manuscript. KH carried out the literature search. All authors read and approved the final manuscript.
